# Population Density and Seasonality Effects on Sin Nombre Virus Transmission in North American Deermice (*Peromyscus maniculatus*) in Outdoor Enclosures

**DOI:** 10.1371/journal.pone.0037254

**Published:** 2012-06-29

**Authors:** Karoun H. Bagamian, Richard J. Douglass, Arlene Alvarado, Amy J. Kuenzi, Brian R. Amman, Lance A. Waller, James N. Mills

**Affiliations:** 1 Population Biology, Ecology, and Evolution Program, Emory University, Atlanta, Georgia, United States of America; 2 Medical Ecology Unit, Viral Special Pathogens Branch, U.S. Centers for Disease Control and Prevention, Atlanta, Georgia, United States of America; 3 Department of Biology, Montana Tech, University of Montana, Butte, Montana, United States of America; 4 Cfwep.Org, Institute for Educational Opportunities, Montana Tech, University of Montana, Butte, Montana, United States of America; 5 Department of Biostatistics and Bioinformatics, Rollins School of Public Health, Emory University, Atlanta, Georgia, United States of America; University of Utah, United States of America

## Abstract

Surveys of wildlife host-pathogen systems often document clear seasonal variation in transmission; conclusions concerning the relationship between host population density and transmission vary. In the field, effects of seasonality and population density on natural disease cycles are challenging to measure independently, but laboratory experiments may poorly reflect what happens in nature. Outdoor manipulative experiments are an alternative that controls for some variables in a relatively natural environment. Using outdoor enclosures, we tested effects of North American deermouse (*Peromyscus maniculatus*) population density and season on transmission dynamics of Sin Nombre hantavirus. In early summer, mid-summer, late summer, and fall 2007–2008, predetermined numbers of infected and uninfected adult wild deermice were released into enclosures and trapped weekly or bi-weekly. We documented 18 transmission events and observed significant seasonal effects on transmission, wounding frequency, and host breeding condition. Apparent differences in transmission incidence or wounding frequency between high- and low-density treatments were not statistically significant. However, high host density was associated with a lower proportion of males with scrotal testes. Seasonality may have a stronger influence on disease transmission dynamics than host population density, and density effects cannot be considered independent of seasonality.

## Introduction

In the past 30 years, numerous theoretical models have been proposed to explain how pathogens become established and spread in host populations. Early models assumed that the driving force behind directly transmitted parasites was population density (density-dependent transmission) and, because these models were useful to understanding many human diseases, they were applied to wildlife populations [1], [2]. For a horizontally transmitted pathogen, higher host population density may lead to higher prevalence of infection, because there is an increased number of potential hosts and because more susceptible hosts provide more opportunities for direct transmission through contact [3]. Additionally, higher densities of infective donors and susceptible hosts may amplify indirect transmission by increasing the amount of infectious pathogen in the environment [4]. Higher host abundance may also result in increased competition for limited resources and mates, increasing stress and leading to decreased immunological capacity [5]. However, the relationship between wildlife host population density and disease prevalence is complex, as reviewed by Adler et al. [3]. While some mark-recapture studies of hantaviruses and arenaviruses in rodent populations in the United States and Europe have indicated a positive concurrent relationship between host population density and infection prevalence [6], [7], others showed an inverse relationship or no direct association [8], [9], [10]. Infection prevalence in wild rodent populations is often associated with host population densities and dynamics in a prior season, an effect known as delayed density-dependent prevalence [11], [12], [13], [14]. For example, regional wild North American deermouse (*Peromyscus maniculatus*; hereafter deermouse) populations in Montana show maximum Sin Nombre hantavirus (SNV) infection (as indicated by antibody prevalence) in the spring, and this peak is often positively associated with the size of the deermouse population the preceding fall [13], [15]. Also, a threshold infection prevalence [13] may be necessary to establish and maintain SNV infection cycles in deermouse populations in Montana. However, some directly transmitted wildlife pathogens display characteristics of frequency-dependent dynamics (where transmission likelihood is independent of population density) [16], [17], or transmission dynamics that vary between density and frequency dependence according to season [18].

The effects of seasonality on disease dynamics in wildlife are another focus of disease ecologists. Seasonal variation in precipitation, temperature, and resource availability can influence host population dynamics, host physiology, and disease dynamics in wildlife host populations [19]. Rodent-borne zoonotic viruses (e.g., hantaviruses, arenaviruses, and cowpox virus) often have seasonal cycles of infection prevalence [6], [7], [14], [20], [21], [22], [23]. Peaks in transmission often coincide with the reproductive season, a time of high social interaction in natural populations [9], [10], [21].

Hantaviruses are directly transmitted, specialist microparasites endemic in natural rodent and insectivore populations; some, including SNV, are pathogenic for humans. Hantaviruses generally establish a persistent infection with long-term shedding in a single natural host species [24], [25]. Because hantavirus infection is chronic, the presence of IgG anti-hantavirus antibody in rodent blood is used as an indicator of active infection. Studies of Old World hantaviruses [e.g. Puumala virus (PUUV)], and the New World SNV and Black Creek Canal virus, indicate that laboratory-inoculated hosts are most infectious and shed the greatest quantity of virus during the acute phase of infection (first 60–90 days) [25], [26], [27]. Although humans primarily become infected by inhaling aerosolized virus from rodent saliva and excreta, the primary route of infection in rodent hosts appears to be via direct contact during aggressive interactions. (i.e., biting and scratching) [10], [28], [29]. Laboratory studies of PUUV indicate that rodent hosts may also be infected via the respiratory route [27], and that PUUV can remain infectious in the environment for up to 15 days [30].

The individual effects of seasonality and density on natural disease cycles are often hard to tease apart from each other and from other confounding factors driving host parasite systems. One way to explore and quantify these effects is through manipulative field experiments using a well studied host-pathogen system. The deermouse-SNV host-pathogen system has been a subject of intensive longitudinal studies that have improved understanding of the relationships between SNV transmission dynamics with seasonal factors and with host population density [10], [31], [32], [33]. Nevertheless, longitudinal studies can be difficult to interpret because of a multitude of confounding factors that characterize uncontrolled, open populations. In addition, a pattern observed at any given time is the product of complex and imperfectly known historical events.

A partial solution to these problems is the use of outdoor, semi-natural enclosures that approximate natural field conditions more closely than does a laboratory. Such studies also allow working with a closed population of a limited number of individuals of known sex, age, physical condition, and infection status, and the events observed during the experiment are largely a consequence of those well known experimental conditions. We used the deermouse-SNV host-pathogen system in Montana to explore the effects of density and seasonality on pathogen transmission. Longitudinal field studies in Montana have demonstrated that the greatest number of seroconversions and the greatest proportion of deermice with detectable SNV RNA are found in the mid-to-late breeding season (June–September; [31], [34]). These data suggest that June–September is the period of greatest virus transmission and, as such, would be the best time to conduct transmission experiments in nature. We conducted 4 transmission experiments using wild, adult, male deermice in outdoor enclosures in Montana during the summer and fall of 2007 and 2008. The enclosure system allowed us to focus on the effects of season and host population density on transmission by controlling for demographic and historic factors, including prior host population densities, by using only adult males and restarting the experiment with new mice or new configurations of mice after 1 or 2 months.

Using mice naturally infected with SNV as donor mice, we tested the hypothesis that the frequency of SNV transmission in deermouse populations is positively correlated with population density, and that this correlation is independent of season. If true, we hypothesized that high-density enclosures would have a greater frequency of transmission events than low-density treatments regardless of when we initiated the experiment. We also explored the influence of season and population density on host reproductive condition, aggressive encounters, and weight gain. In this paper, we focused on ecological, behavioral, and physiological aspects of host population density and seasonality as they relate to SNV transmission. In a second paper, we will focus on the molecular and immunologic aspects of transmission including time course of infection and differences among individual hosts.

## Materials and Methods

### Ethics Statement

All animal work was conducted according to relevant national and international guidelines. All components of this study were reviewed and approved by the appropriate institutional animal care and use committees (Emory University IACUC protocol #D10-1109-02R07, U.S. Centers for Disease Control and Prevention IACUC protocol #1500MILRODX-A1, and University of Montana IACUC protocol #AUP 009-07). The study was also reviewed and approved under Emory University Biosafety protocol #100-2008. No trapping permit is required for trapping rodents in Montana.

### Study Site and Enclosure Construction

**Figure 1:**
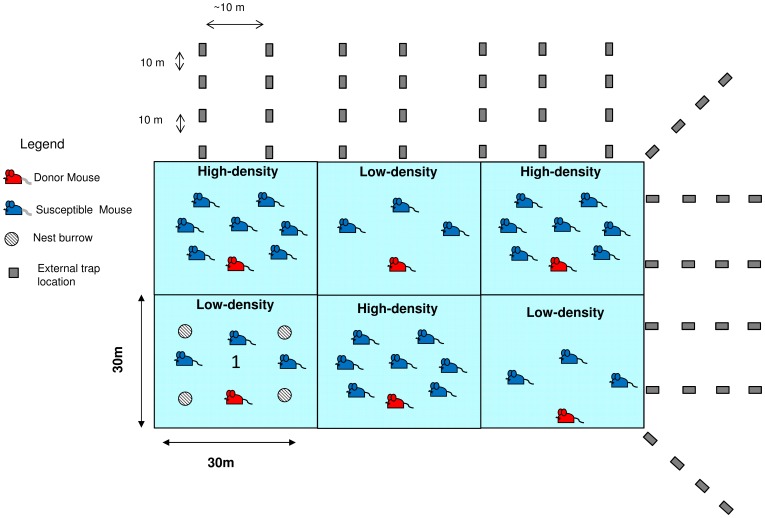
Diagram of enclosures, nest burrows, and experimental design for density experiments. Each enclosure had 4 nest burrows as depicted in Enclosure 1 (lower left). The external trapping grid had 26 lines of traps in 4 rows; traps were spaced approximately 10 meters apart (farther at the corners; drawing not to scale). The first trap of each line was placed flush to the enclosure, with all subsequent traps spaced about 10-m apart. Although the external grid surrounded the entire enclosure array, only two sides are depicted. Figure applies to experiments 1–3. Experiment A differed in having 3 susceptible mice in all 6 enclosures (i.e. no high density treatment).

This study was conducted in shrub-steppe grassland near Butte, Montana, USA, May–October 2007 and August–September 2008. We conducted 4 experiments–1 preliminary transmission experiment (experiment A) and 3 density experiments (experiments 1, 2, and 3; Table 1, Figure 1). Experiments were run in 6, 0.1-ha enclosures constructed of sheet metal [35], [36], with walls extending approximately 1 m above ground and 0.6 m underground. Each enclosure contained 4 underground nest burrows [37] that provided safe, permanent cover for the mice. Within each enclosure, we placed 36 trapping stations approximately 4 m apart. One Sherman live-capture trap (H. B. Sherman Traps, Tallahassee, Florida, USA) was placed at each trap station for up to 3 consecutive nights (until all mice were captured) weekly or biweekly, depending on the experiment. Traps were baited with peanut butter and rolled oats, and contained polyester Fiberfil bedding. See Figure S1 & S2 for photographs of the study site, and Appendix S1.1 for detailed descriptions of the habitat, enclosure protocols, and nest burrows.

**Table 1:** Experimental design and transmission events per experiment and density treatment for SNV transmission experiments in deermice in outdoor enclosures near Butte, Montana, 2007–2008†.

Exp	Season	Dates	Type of Exp	Duration (weeks)	Sampling Frequency	Susceptible mice per enclosure	Transmission Events	Total Susceptible^a^	Total Susceptible (adjusted)^b^
EXP A	Early Summer	Jun 11–Jul 09 07	Transmission	4	2 weeks	3	6	18	17^c^
EXP 1	Mid- Summer	Jul 17–Aug 23 07	Density	5	2 weeks	3; low	3: high	30	24^d^
						7; high			
EXP 2	Late Summer	July 24–Sept 25 08	Density	8	1 week	3; low	6: high	30	33^e^
						7; high	2: low		
EXP 3	Fall	Sept 03– Oct 16 07	Density	6	2 weeks	3; low	1: high	30	27^f^
						7; high			

Exp = experiment; High  =  high density treatment; low  =  low-density treatment.

† Each enclosure had one donor mouse.

a See Figure 1.

b Adjusted; number of susceptible mice used to calculate transmission incidence.

c Excludes 1 mouse never recaptured after initial release into the enclosure.

d Excludes 3 mice never recaptured after initial release, 2 susceptible mice from low-density enclosure in which the infection status of donor was unclear, and 1 mouse which we cannot rule out as being exposed prior to release in the enclosures.

e Includes 3 substitute susceptible mice that were released into the enclosures to replace dead mice and 1 escapee to keep population densities constant.

f Excludes 3 mice never recaptured after initial release into the enclosure.

### Experimental Design

For each experiment, 1 infected (donor) and a predetermined number of uninfected (susceptible) mice were released into each enclosure according to the study design (Table 1 and Figure 1). As we were trying to simulate low and high deermouse population density conditions in Montana, we used a 15-year mark-recapture dataset from Montana to determine our experimental treatments. We have observed high-density populations that have been consistently around 100–120 deermice/hectare at a few of our sites [38], so 80 deermice/hectare is not unnaturally high. We chose our low population density based on available data from areas around our study site [39] and others [38]. Although population densities can be lower than the 40/ha that we used, we also had to maintain populations large enough to achieve statistical power. If we used 20/hectare (we would be releasing just 1 donor and 1 susceptible mouse into an enclosure), it would have been impossible to conduct meaningful statistical tests. We alternated the enclosures housing the low- and high-density populations at each repetition of the experiment. During Experiment 2, we replaced 1 donor and 3 susceptible mice that died (carcasses were recovered) and 1 enclosure mouse who was captured outside the enclosure, with additional quarantined susceptible and donor mice to maintain constant population densities throughout the experiment.

All mice released into the enclosures were ear-tagged with sequentially numbered, metal, fish fingerling tags (National Band and Tag Company, Newport, Kentucky, USA). Mice were provided grain, apple chunks, and water weekly or as needed. Food was scattered widely throughout the enclosure to avoid unnatural aggregations at feeding stations; water was provided in water-bottles, as required by our IACUC protocol, in the burrows. Rodents in enclosures were trapped weekly (2008) or biweekly (2007) to collect blood samples using standardized protocols for SNV surveillance [40]. Mice were handled and sampled according to strict guidelines developed by the U. S. Centers for Disease Control and Prevention and designed to prevent cross contamination between rodents and infection to humans [41]. Blood samples were immediately frozen on dry ice and stored at −70°C until processing. Body weight, breeding condition (scrotal or abdominal testes), trap location, and number of wounds on the ears and tail (as an indicator of aggressive encounters) were recorded during each trapping session. We tested all blood samples collected from all experimental animals for SNV RNA and antibody as described (Appendix S1.2). We also constructed a 0.5-ha trapping grid outside of the enclosures (see Figure 1), and tagged and released the outside mice to monitor non-experimental rodent population dynamics and to detect any escapees during each experimental run. While outside rodents were trapped and monitored for escapees for the entire duration of each experiment, descriptive data were collected for the majority, but not all, trap sessions (until September 19, 2007, and until September 4, 2008) due to personnel constraints.

### Rodent Collection and Selection of Experimental Subjects

Mice trapped within 5 km of the study area were assigned to 1 of 3 age classes according to body weight: mice <14 g were juveniles; mice 14–17 g were subadults; mice >17 g were adults [10], [40]. Testes position (scrotal vs. abdominal) was used to determine breeding condition. We selected adult, male mice, to eliminate demographic factors such as sex and age from our experiments, and because adult males are responsible for the majority of SNV transmission in wild populations [9]. In the event that there were not enough adult males captured, we included larger subadults and made sure that the age structure of the experimental mouse populations was as similar as possible among enclosures. Because genetic relatedness might influence social interactions and immunological responses to infection, we avoided placing mice from the same capture site within the same enclosure. Sin Nombre virus infection status of mice was determined by detecting IgG antibody [42], [43] and by detecting SNV RNA by nested RT-PCR [44]. In 2008, susceptible mice were quarantined prior to release into the enclosures, while in 2007 they were not. See Appendices S1.2 and S1.3 for details of testing, quarantine, and selection of susceptible animals.

Rodent hosts of other hantaviruses are most infectious 2–5 weeks post-infection, but are known to shed infectious virus for much longer [45], [46], [47]. In 2007, we chose donor mice as those positive for SNV RNA or antibody. In 2008, the quarantine period allowed us to choose recently seroconverting mice (see Appendix S1.3).

### Transmission Event Mice

After the start of each experiment, if a susceptible mouse in an enclosure was found positive for either SNV RNA or SNV antibody, he was designated as a transmission-event (TE) mouse. Every TE mouse was found positive for both SNV RNA and antibody, except for 3 mice that did not develop detectable IgG antibody before either dying or the end of experiment. For these 3 mice, we confirmed infection with SNV by detecting SNV RNA in 2 or more blood samples collected on different dates, or by sequencing the samples (2008 mice).

Because the mice in 2007 were not quarantined, it is possible that some were infected prior to release into the enclosures. The majority of TE mice had negative SNV antibody and RNA results for at least 2 weeks post-release and seroconverted or had detectable SNV RNA in their blood 1 month post-release. Our 2008 quarantine results indicated that mice that were previously exposed seroconverted within the first 2 weeks. Three of the TE mice seroconverted within two weeks after introduction into the enclosures. Two of these three mice had very low antibody titers (Mouse 1: titer of 100, no RNA results (not enough blood available for test), Mouse 2: titer of 200, positive for SNV RNA), which is consistent with the blood profile of a very recently infected mouse (Bagamian 2012). Also, both mice were from the same enclosure, suggesting close temporal exposure to the same donor. Fifteen of the 18 transmission events (excluding these two mice: 13/16) involved multiple mice in the same enclosure (Bagamian 2012). Thus we feel that infection prior to release into the enclosures is unlikely for these two mice.

Nevertheless, we analyzed our data both including and excluding these mice and report both sets of results. A third mouse was SNV RNA-positive and had a high antibody titer (1600) two weeks post-release into enclosures. Because infection prior to release into the enclosure seemed possible, this mouse was excluded from our analyses of transmission incidence. Although we feel that these criteria for excluding potential transmission events prior to the experiment are reasonable, we emphasize that we have no way of excluding such events with 100% certainty. We report transmission incidence as the number of new infections (transmission events/sum of the number of mouse-weeks of observations) (see Appendix S1.4). For more details regarding TE mice, see Appendix S1.3.

### Statistical Analyses

We conducted statistical analyses (Fisher’s exact tests, tests of differences between proportions, t-tests, and simple linear regression) using Microsoft Excel 2007 and R (R Development Core Team, Vienna, Austria, 2011). Details of the analyses and variable derivation are provided in Appendix S1.4.

For all analyses, we excluded data from mice that were released into the enclosures and never recaptured (n = 7 for 2007 experiments; n = 1 for the 2008 experiment). We also excluded data from 1 low-density enclosure (Enclosure 3) in Experiment 1 in analyses of transmission incidence, because it was unclear whether the donor mouse was truly infected. His blood was positive for SNV RNA in 1 of 2 samples, but he was not recaptured again to reconfirm infection status. There were no TE mice in Enclosure 3 in Experiment 1. In analyses of wounds, scrotal condition, and weight gain for the 2008 experiment, we also excluded information from 2 mice that were in the experiment for less than 2 weeks, because of insufficient data.

### Wounding

We analyzed the total number of new wounds on each mouse per experiment, season, or density treatment. The total number of new wounds was counted on an individual animal over the course of the experiment, and each animal was represented only once in any analysis. This conservative measure only includes wounds detected on a new location on the mouse (tail vs. ear) and increases in the number of wounds from the previous sampling session. This ensured that the same wound was not counted twice for any animal, but also allowed for the possibility that some new wounds in the same area as a previous wound may not have been counted. We ran a linear regression model with the season as a categorical predictor variable, and the outcome variable was the number of new wounds per experiment.

## Results

### Relationship of Incidence to Seasonality and Density

**Figure 2:**
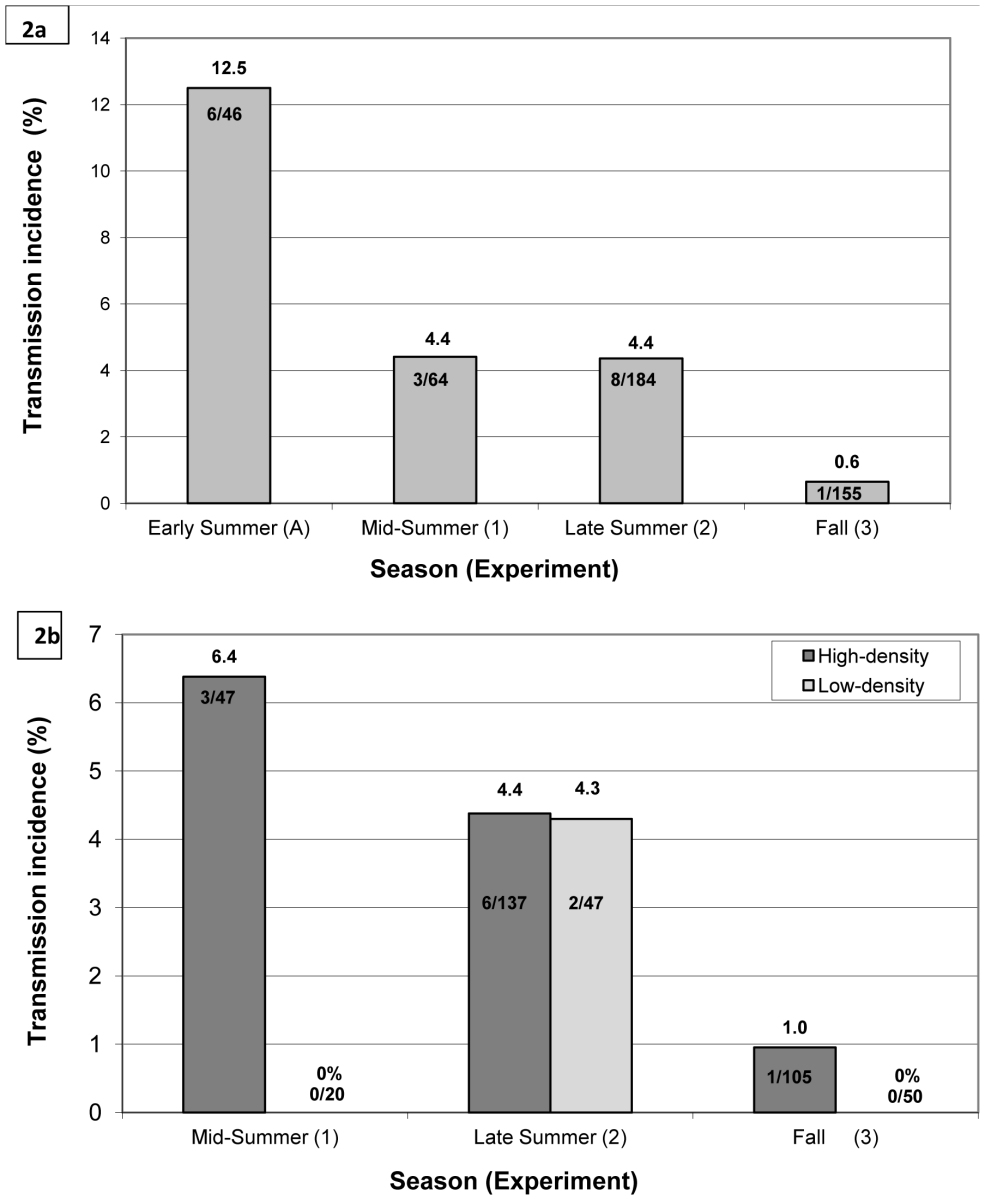
Incidence of Sin Nombre virus transmission in North American deermice *Peromyscus maniculatus*). (a)incidence by season/experiment and (b) incidence by density treatment and experiment. The incidence of transmission (number of transmission events per 100 mouse-weeks of observation, expressed as a percentage (see [20], Appendix S1.4) is reported above the each bar for (a) each season (each experiment A, 1, 2, 3) and (b) per density treatment for experiments 1–3. Numbers of transmission events/mouse-weeks are reported within each bar.

We documented 18 transmission events over 4 experiments (Figure 2a). Because the susceptible mice in 2007 were not quarantined prior to release into the enclosures, it is possible that some were infected prior to release into the enclosures (see discussion in Materials and Methods above). The transmission incidence was not significantly different between the high- and low-density treatments combining data from all 3 density experiments (z = 0.91, p = 0.37) or within each experiment, according to the test of differences between proportions (Exp. 1: z = 1.15, p = 0.25; Exp. 2: z = 0.023, p = 0.98; Exp. 3: z = 0.71, p = 0.48; see Figure 2b for transmission incidences). The proportion of TE mice to overall susceptible mice was not significantly different between high- and low-density treatments overall (two-tailed Fisher’s exact test [FET]: p = 0.34) and within each experiment (FET: Exp. 1: p = 0.54, Exp. 2: p = 1.00, Exp. 3: p = 1.00).

We found statistically significant differences in the incidence of transmission between each summer period (early, mid, late) and the fall demonstrated by both rate ratio confidence levels and by the test of differences of proportion including all 18 transmission events, and when the 2 potentially questionable events were removed from the analyses (Table 2). Transmission incidence during the summer months ranged from about 7 (late summer) to 19 (early summer) times greater than the fall when all transmission events were included in the analyses, and from about 7 (late summer) to 13 (early summer) times greater when the two events were removed (Table 2). Incidence in mid-summer and late summer was 1/3 of the incidence in early summer when all transmission events were considered and when the two transmission events were removed, incidence in mid- and late summer was half the incidence in early summer (Table 2). These within-summer differences were significant by rate ratio confidence intervals. According to the test of differences of proportion, the only statistically significant within-summer comparison was between the incidences in early summer and late summer (z = 1.78, p = 0.04), when all transmission events were considered (Table 2). The proportion of TE mice to overall susceptible mice in the fall was significantly lower than in the early summer (FET: p = 0.01) and late summer (FET: p = 0.03), but not in mid-summer (FET: p = 0.4; Table 2), when all transmission events were considered (Table 2; see Table 1 for mouse numbers). All other comparisons of proportion of TE mice to overall mice between experiments were not statistically significant by FET (p>0.1 for all comparisons), when all transmission events were considered. When two transmission events were removed from the analyses, the proportion of TE mice to overall susceptible mice in the fall was still significantly lower than in the early (FET: p = 0.046) and late summer (FET: p = 0.03) (Table 2); all other comparisons were not significant (FET: p>0.15).

**Table 2:** Seasonal transmission incidence ratios for SNV transmission experiments.

Season	Seasonal Comparison	Rate Ratio, (95% CI)
**All reported transmission events**		
Early summer**	Fall	**19.38, (14.02–26.78)**
Mid-summer*	Fall	**7.27, (5.43–9.72)**
Late summer**	Fall	**6.76, (5.45–8.37)**
Mid-summer	Early Summer	**0.38, (0.26–0.55)**
Late Summer*	Early Summer	**0.35, (0.25–0.48)**
Late Summer	Mid-summer	0.93, (0.70–1.24)
**Excluding two transmission events from early summer**		
Early summer**	Fall	**13.48, (9.70–18.73)**
Mid-summer*	Fall	**7.27, (5.43–9.72)**
Late summer**	Fall	**6.76, (5.45–8.37)**
Mid-summer	Early Summer	**0.54, (0.37–0.79)**
Late Summer	Early Summer	**0.50, (0.36–0.69)**
Late Summer	Mid-summer	1.07 (0.81–1.42)

Relative ratios for each pairwise comparison between seasons. Season used as numerator in rate ratio is listed first. Statistically significant rate ratios and confidence intervals are in boldface type.

* Seasonal comparison statistically significant by test of difference of proportions.

** Seasonal comparison statistically significant by Fisher’s exact two-tailed test and test of difference of proportions.

### Relationship of Wounding to Seasonality and Density

**Figure 3:**
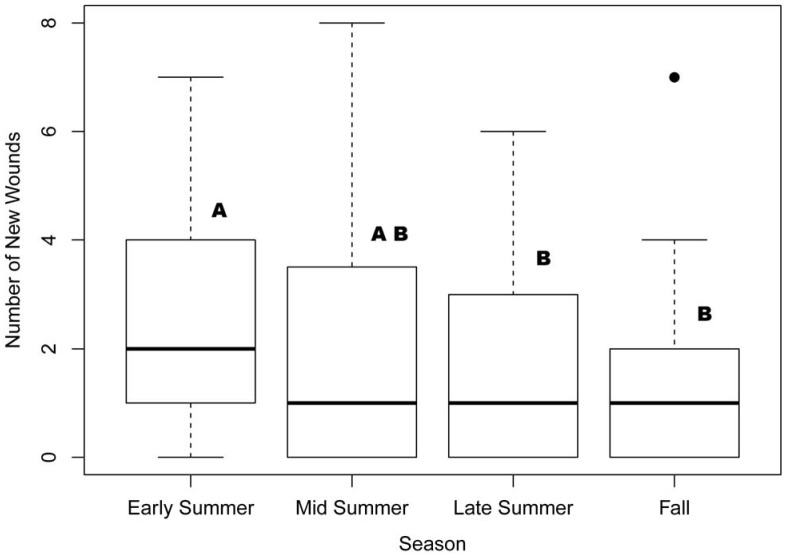
Seasonal median number of new wounds per individual deermouse. Thick horizontal line is the median; top and bottom of boxes represent the 25th and 75th percentiles; whiskers indicate ranges, excluding outliers. Outlier is indicated by black dot. Medians with the same letter above the box are not significantly different.

The average number of new wounds per mouse in the early summer was significantly higher than in the fall (t_103_ = −1.998, p  = 0.048, β_fall_  = −1.1522, SE = 0.5767), and suggestively higher in comparison to late summer (t_103_ = −1.946, p = 0.054, β_LateSummer_  = −0.9943, SE = 0.5109), but not in comparison to mid-summer (t_103_ = −1.525, p>0.05), as determined by a linear regression comparing each season to early summer (Figure 3).

We found no significant differences in the average number of new wounds on individual mice between high- and low-density treatments overall (high-density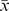 = 1.85, SD = 1.80; low-density: 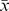 = 1.58, SD = 1.97; t_90_ = −0.656, p>0.05) or in each experiment (p>0.15 for all within experiment comparisons).

### Relationship of Host Reproductive Condition and Weight Gain to Seasonality and Population Density

**Figure 4:**
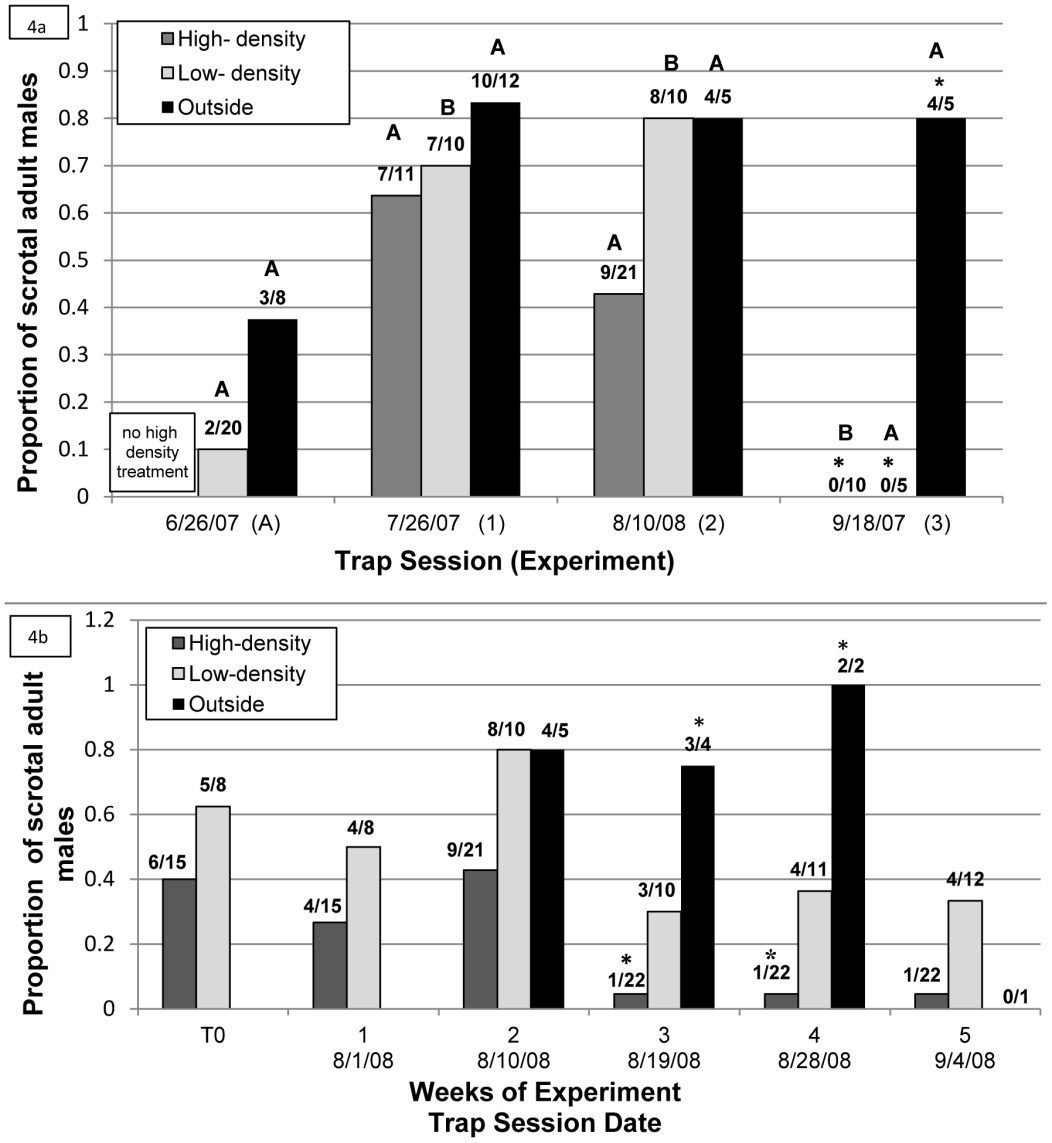
Proportion of adult, male deermice with scrotal testes inside and outside enclosures. a) The proportion of scrotal adult males (of total adult males captured) 2 weeks post-release into the enclosures inside (by density treatment) and outside of enclosures during each experiment. Experiment A only had low-density treatment groups; experiments 1–3 had high- and low- density treatments. Numbers of scrotal/total for each experiment are denoted above bars. Bars with the same letter above them are not significantly different within each category (high, low, outside) between experiments. Statistically significant comparisons between categories (high vs. low vs. outside) for experiment 3 are indicated by asterisks. b) Proportion scrotal at 4 trapping sessions by density treatment and by location (inside vs. outside enclosures) during Experiment 2. Numbers of scrotal/total for each trap session are denoted above bars. Statistically significant differences between categories (high, low, outside) at a given trap session are indicated by asterisks.

The proportions of adult males with scrotal testes in the enclosures varied by season of the experiment (Figure 4a). In the low-density treatments, the proportion of males with scrotal testes 2 weeks post-release was significantly higher in the mid-breeding season (July and August) than either the early season (FET: June vs. July: p = 0.002, June vs. August: p<0.001) or the late season (FET: September vs. July: p = 0.03, September vs. August: p = 0.01). In the high-density treatments, the proportion of males with scrotal testes was significantly lower at the end of the breeding season (September) than in the mid-breeding season (FET: September vs. July: p = 0.003, September vs. August: p = 0.03). No other tests for the proportions of males with scrotal testes between capture dates were significant (FET: p>0.45). The proportions of adult males with scrotal testes captured outside the enclosures (Figure 4a) did not differ significantly between months (FET: p>0.05).

Two weeks post-release into the enclosures, no differences were observed in the proportion of males with scrotal testes between the low- and high-density treatments (Figure 4a) in any of the 3 density experiments (FET: p>0.06 for all comparisons). However, in Experiment 2, the longest running experiment (8 weeks; Figure 4b), mice from the low-density group, on average, remained scrotal significantly longer than mice from the high-density group (4.16 weeks vs. 1.6 weeks; t_37_ = −4.04, p<0.001). The proportion of scrotal males in the low-density group was generally higher than that of the high density population, and lower than the outside mouse populations, but none of these differences were significant (FET: p>0.19; Figure 4b). The high-density population had a significantly lower proportion of scrotal males as compared to the outside population on the third and fourth trap session (FET: August 19, 2008: p = 0.030, August 28, 2008: p = 0.048; Figure 4b). No significant differences were observed in the proportions of males with scrotal testes by density treatment at the start of experiment 2 (Figure 4b).

Pooling data across all experiments, 2 weeks post-release, the proportion of scrotal males with wounds (77%) was significantly higher than the proportion of abdominal males with wounds (54%; FET: p = 0.03). Wound frequency did not differ significantly between adult males in the enclosures compared to those captured outside the enclosures (FET: p = 1.00).

When measured at the beginning of each experiment and at the end of each experiment, no statistically significant differences were found in the mean animal weight between treatments (high- vs. low-density) or between locations (inside vs. outside enclosures) in any of the experiments (p>0.20 for all comparisons). During Experiment 2, there were no significant differences between treatments in the mean rate (g/week) of weight gain (low-density: 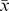 = 0.300, SD = 0.343; high-density: 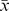 = 0.279, SD = 0.372; t_35_ = 0.1675, p>0.05).

## Discussion

Our objectives were to observe natural transmission of SNV in *P. maniculatus* populations in a semi-controlled outdoor setting, to empirically test the influence of seasonality and density on the frequency of transmission in a closed population, and to clarify the relationships between seasonality, density, host aggression, and reproductive physiology. According to theoretical models and mark-recapture data, all of these factors influence disease dynamics, but we are the first to examine these variables by experimentally manipulating host population densities across seasons in a field setting. The high-density group had many more SNV transmission events than the low-density group (11 and 2, respectively). Transmission of SNV in low-density enclosures occurred in only 1 of the 3 density experiments, and the overall transmission incidence in the high-density treatment was 2-fold higher than in the low-density treatment. Nevertheless, this difference between the density treatments was not statistically significant. Sample sizes were low and any effect of density on transmission frequency might have been obscured by the number of observed zeros. Alternatively, SNV transmission may be frequency dependent. Six years of cowpox virus dynamics in bank voles showed that transmission appeared to be density dependent during the winter, but frequency dependent in the summer–emphasizing the importance of seasonal variation in host behavior and susceptibility on disease processes [18]. As we found a strong effect of seasonality in our experiments, it is possible that underlying SNV transmission processes may exhibit similar variations. A larger sample size and more iterations of the experiment over a longer range of seasons may be needed to more reliably quantify these apparent differences. Unfortunately, large-scale enclosure experiments are very time consuming and labor intensive, and require a large area and much construction material.

The incidence of SNV transmission decreased significantly as experiments were conducted later in the breeding season. This observation is consistent with previous mark-recapture studies, which have indicated strong seasonal trends in seroconversion and increased prevalence of infection during the breeding season [10], [31], [34]. Douglass *et al.* [34] reported that the incidence of seroconversions remained relatively high but constant throughout the breeding season, while we detected a decreasing incidence from June to October. However, that study reported seroconversions detected at monthly sampling intervals in free-roaming populations across Montana; we detected transmission events weekly or biweekly and were able to assign a tighter temporal window to the events.

Initiation and cessation of the breeding season for *P. maniculatus* populations are highly variable and depend on photoperiodic cues, temperature, and food availability [48]. These influential factors vary geographically and annually, and may trigger differential effects among individuals in the same population [48]. At our site, the proportion of adult males in breeding condition captured in the enclosures was significantly greater in experiments conducted during the mid-breeding season than in the early and late breeding season. Also, fewer scrotal males were captured outside the enclosures in the early breeding season than in the mid-breeding season, although this trend was not statistically significant (Figure 4a). This pattern differs from previous reports from longitudinal data in southwestern and central Montana, where the percentage of scrotal males often peaked at 80% during May or June, and decreased linearly over the course of the breeding season to approximately 2% in October [10]. Our analyses included only adult males, but in the open population studied by Douglass *et al.* [10], the proportion abdominal would have continuously increased throughout the breeding season through the recruitment of young of the year.

Studies of caged albino and wild-type house mice, free-roaming vole populations (*Microtus montanus* and *Microtus pennsylvanicus*), and *P. maniculatus bairdii* have shown a strong and significant effect of high population densities in suppressing reproduction in both males and females [49]. In all of these species, in animals living in densely populated areas, there was an increased investment in adrenocortical-related glands, but little or no gonadal development or function [49]. The adrenocortical response assists in survival when individuals are faced with extreme environmental changes or physiological stress [49]. Although the deermouse population density in our high-density enclosures (80 mice/ha) was similar to naturally observed high population densities in Montana, this density appears to be sufficient to affect the reproductive function of these mice. At most trapping sessions, the proportion of reproductive males in the low-density group was similar to that in the outside population. The population density of male and female mice outside the enclosures ranged from 28–46 mice/ha in August 10–28, 2008, which was similar to our low-density treatment (40 mice/ha). Although the majority of comparisons were not statistically significant between density treatments, in 2 of the 3 density experiments, the high-density enclosures consistently had lower proportions of reproductive adult males than low-density enclosures (Figures 4a & b). In Experiment 2, the percentage of adult males in breeding condition in high-density enclosures decreased from 40% to 5% during the third week of the experiment, and remained at that low level, while in the low-density enclosures, that percentage remained consistently around 30% (Figure 4b). Also, mice from the low-density group were in reproductive condition significantly longer than the mice from the high-density group in Experiment 2. When data were pooled across experiments 1–3, the proportion of adult males with scrotal testes was significantly lower in the high-density group than in outside mice (FET: outside vs. high-density: p = 0.01, outside vs. low-density: p = 0.57). This suggests that the decrease in reproductive condition was primarily a result of high population density. Although our experiment does not provide sufficient data to test such a hypothesis, we speculate that the decrease in sexual preparedness associated with high density conditions may result in decreased aggression, improved immune system function, and potentially decreased incidence of transmission. This might help explain some of the difficulty in demonstrating a clear positive relationship between population density and SNV transmission.

Independent of any treatment effects of density or season, the enclosure may have affected the length of time mice remained scrotal. When data from all 4 experiments were pooled, a significant decrease in the overall proportion of scrotal males emerged during the first trap session (Time 1: T1) after release in comparison to before they were released into the enclosures (Time Zero: T0; FET: p = 0.047; data not shown). The proportion of scrotal adult males in the enclosures at T1 was also significantly lower than the proportion of scrotal adult males captured outside the enclosures (FET: p = 0.003, data pooled across all 4 experiments). Additionally, in the second-longest running experiment (Exp. 3; 6 weeks), while approximately 30% of males in the high-density group and 10% of males in the low-density group had scrotal testes at T0 (data not shown), no males with scrotal testes were captured at T1 and at the next 2 trapping sessions, although breeding males were captured outside the enclosures at T1 (see Figure 4a). One important factor may have been the absence of females inside the enclosures. Approximately 8–14 female mice were consistently captured outside, and most were pregnant or in breeding condition. However, despite the absence of females, 3 of the 4 experiments (except for the final fall experiment) always contained males in breeding condition, indicating the importance of seasonal cues in influencing breeding cycles. Additionally, enclosed males may have still received olfactory cues from nearby females outside the enclosure.

Although population density clearly affected the ability to maintain breeding condition, it had no statistically significant effect on the rate of weight gain. The supplementary food and water in the enclosures may have contributed to weight maintenance.

The average number of new wounds per mouse was significantly higher in the early summer than late summer and fall. As the breeding season begins, males often respond to seasonal cues and establish and defend territories [48], leading to increased wounding. The higher prevalence of wounds on males with scrotal testes supports the idea that breeding males are more likely to be aggressive and interactive than non-breeding males. At the end of the breeding season (late summer- early fall; [10]), there are fewer breeding males, and, therefore, fewer fights.

The fact that incidence of transmission and average number of new wounds per mouse peaked at the beginning of the breeding season and decreased over time provides some support to the current view that direct contact may be the primary mode of transmission in wild deermouse populations, because the most transmission occurred during times where the mice were most aggressive. We cannot rule out the possibility that SNV may have been transmitted both directly and indirectly in the enclosures. Future studies could implement cameras, pit tag recorders, and fluorescent marking powder [50] to gather a better understanding of the contact structure and dominance dynamics within enclosed populations and their relationship to transmission dynamics. Future manipulative experiments in enclosures will also allow testing hypotheses that environmental transmission may occur in nature.

A major limitation of our experiments was small sample size. We were able to maintain a limited number of mice per enclosure, and we observed 18 transmission events total in all 4 experiments. However, as natural transmission events are rare by nature, recording 18 events in a semi-controlled setting could be considered very successful. A previous laboratory study reported only 1 SNV transmission event out of 54 attempts [51]. Nevertheless, larger experiments with greater numbers of mice per enclosure and increased numbers of replicate enclosures would have greater statistical power. Also, as we did not quarantine our susceptible mice after our experiments, we may have underestimated transmission rates. We conducted our experiments during only 2 seasons (summer and fall). To more completely understand seasonal effects on this system, subsequent studies should be run in winter and spring. Such studies may be challenging (especially in Montana) because of weather conditions and presumably decreased transmission during these seasons, although transmission during winter huddling in nest boxes could be examined. We also did not control or test for genetic variability in resistance to infection or dominance hierarchies, factors that may have influenced infection dynamics within the enclosures. Finally, in order to decrease the number of variables and keep our experiment simple and most likely to succeed, we used only male deermice. We do not know how this unnatural condition may have affected our results. Male-female mixed populations are a more natural arrangement of hosts, and therefore, to more fully understand natural SNV transmission, future experiments should also be conducted using mixed male and female populations. Comparing and contrasting the transmission and behavioral dynamics between same-sex and opposite-sex arrangements may help elucidate the relative roles of each type of interaction in disease transmission in the wild.

Our results, especially in the light of previous mark-recapture studies of effects of season and density on infection dynamics in wildlife populations, emphasize the importance of considering the strong effects of season as a confounder when making comparisons of density effects in natural populations. Seasonality, even when only evaluated within the timeframe of the breeding period (spring to autumn), may be more influential in disease dynamics than population density. Season influences host behavior, susceptibility, host reproduction, and other physiological processes, all of which are critical in maintaining disease transmission cycles in nature. Although there is a likely effect of host population density on disease transmission, density processes cannot be considered independently of seasonal factors when exploring natural host-pathogen systems.

We successfully conducted large-scale manipulative experiments that followed SNV transmission in deermice under controlled conditions. Our experiments provided further insight into the effect of seasonality and density on hantavirus transmission, reservoir host aggression, and host reproductive processes. Our successful methodologies might be used to address other questions in the field of wildlife disease ecology or in similar zoonotic host-pathogen systems.

## Supporting Information

Figure S1 **Sheet metal enclosure array used for containing deermice in Montana.**
(JPG)

Figure S2 **Interior corner of one enclosure showing Sherman trap.**
(JPG)

Appendix S1 **Supplementary methods for Population Density and Seasonality Effects on Sin Nombre Virus Transmission in North American Deermice (** ***Peromyscus maniculatus*** **) in outdoor enclosures.**
(DOCX)
