# Supplementary figures and images for: Population Density and Seasonality Effects on Sin Nombre Virus Transmission in North American Deermice (Peromyscus maniculatus) in Outdoor Enclosures
Source: PLoS One. 2012 Jun 29;7(6):e37254. doi: 10.1371/journal.pone.0037254 (PMC3387171; doi:10.1371/journal.pone.0037254)

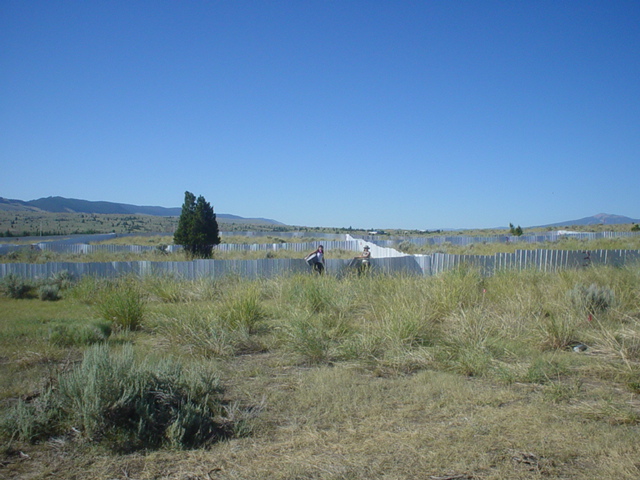

Supplement: Figure S1 — Sheet metal enclosure array used for containing deermice in Montana. (JPG) [file pone.0037254.s001.jpg]

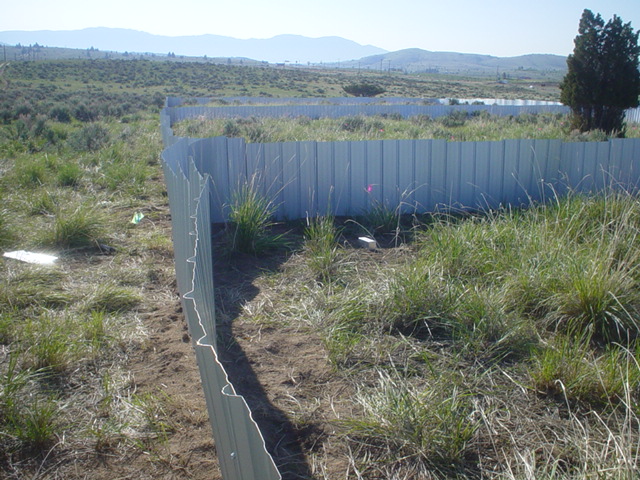

Supplement: Figure S2 — Interior corner of one enclosure showing Sherman trap. (JPG) [file pone.0037254.s002.jpg]
