# Supplementary material for: Population Density and Seasonality Effects on Sin Nombre Virus Transmission in North American Deermice (Peromyscus maniculatus) in Outdoor Enclosures
Source: PLoS One. 2012 Jun 29;7(6):e37254. doi: 10.1371/journal.pone.0037254 (PMC3387171; doi:10.1371/journal.pone.0037254)
Supplement: Appendix S1 — Supplementary methods for Population Density and Seasonality Effects on Sin Nombre Virus Transmission in North American Deermice ( Peromyscus maniculatus ) in outdoor enclosures. (DOCX) [file pone.0037254.s003.docx]

**Appendix for**

**Population Density and Seasonality Effects on Sin Nombre Virus Transmission in North American Deermice (*Peromyscus maniculatus*) in Outdoor Enclosures**

Karoun H. Bagamian, Richard J. Douglass, Arlene Alvarado, Amy J. Kuenzi,

Brian R. Amman, Lance A. Waller & James N. Mills

**Methods**

- 1. **Nest burrow and enclosure descriptions and protocols**

The enclosures were built in a shrub-steppe habitat. The vegetation inside the enclosures consisted of a mixture of Rocky Mountain juniper (*Juniperus scopulorum*), big sagebrush (*Artemisia tridentata*), rubber rabbitbrush (*Chrysothamnus nausiosus*), Idaho fescue (*Festuca idahoensis*), blue bunch wheatgrass (*Agropyron spicatum*), and Canada bluegrass (*Poa compressa*). To impede escapes from the enclosures, all vegetation within 1 meter of the enclosure walls, both inside and outside of the structure, was mowed, and any branches or vegetation hanging or growing in the 1-meter perimeter were cut or removed before and during the experiment.

Each enclosure had 4 evenly spaced nest burrows. Each nest burrow consisted of a 20.3-cm diameter polyvinyl chloride (PVC) pipe nest chamber that was 45.7 cm tall, with a 2.5-cm, cement floor. Each burrow had a 2.5-cm diameter PVC pipe entrance tunnel set at a 45 degree angle. They had insulated lids that consisted of a 4 × 28-cm wooden board affixed with circular pieces of Styrofoam, and a galvanized metal lid [[1](#_ENREF_1)]. Nest burrows were buried so that the top was flush with the surface of the soil and the insulation fit inside the top 2 inches of the nest burrow, with the roof extending over the surface of the soil. The entrance to the nest burrow had small rocks arranged to keep rain water out. A water bottle was wired to the bottom of each nest burrow lid and replenished when almost empty. Scratch grain and apple chunks were scattered within the enclosures weekly or as needed.

We checked enclosure perimeters every 1–2 days and performed repairs as necessary. Nest burrows were cleaned weekly during the experiments by removing all nesting materials and feces. Between experiments, they were thoroughly cleaned of nesting materials and any feces and cached food, sprayed with virucide, and left with the lids off under direct sunlight for several days to dry and inactivate any residual infectious virus. During testing of blood samples, we housed mice in individual, homemade 35 X 35 X 55-cm tall mouse boxes with screw-top lids and ventilation holes around the top.

**1.2 Immunological and molecular testing**

We collected blood samples from the submandibular vein using a Goldenrod lancet (Medipoint International, Inc., Mineola, New York, USA) or by capillary tube from the retro-orbital capillary plexus after anesthesia with isofluorane. In the laboratory, we tested blood samples for IgG antibody reactive with SNV recombinant nucleocapsid protein (supplied by the U.S. Centers for Disease Control and Prevention [CDC], Atlanta, Georgia, USA) by enzyme-linked immunosorbent assay (ELISA) according to standard protocols (some 2007 blood samples) or by a rapid peroxidase enzyme-linked immunosorbent assay (PAGEIA) (blood samples from 2007 and 2008) [[2](#_ENREF_2),[3](#_ENREF_3)]. All 2007 blood samples positive for anti-SNV antibody by ELISA were also tested by PAGEIA, and the results were identical. We determined antibody titers using the PAGEIA assay. The blood samples were initially diluted 1:100 in phosphate buffered saline and then serially diluted in a log2 series from 1:1,000 to 1:128,000. Both the ELISA and the PAGEIA detect IgG antibody to most or all New World hantaviruses, but do not distinguish among them. However, we confirmed all hantavirus IgG antibody-positive blood samples by molecular testing. We tested blood samples specifically for SNV RNA using reverse transcriptase-polymerase chain reaction (RT-PCR) as described previously [[4](#_ENREF_4)]. Mice were housed 1 per plastic mouse box until blood analyses were complete. We employed extra precautions to prevent cross-contamination during caretaking duties and storage of mice (using clean gloves while replacing feed and water, keeping boxes spaced about a foot apart, etc.), and we observed no evidence of cross contamination.

**1.3 Selection of susceptible and donor animals**

In the 2007 experiments (Experiments A, 1, 3), we designated rodents as susceptible if they had no detectable SNV RNA by RT-PCR and no detectable IgG antibody to SNV in blood. Except for the time between capture and testing results (4–10 days), these mice were not quarantined prior to release into the enclosures for each 2007 experiment. Any mouse positive for SNV RNA or antibody was considered as a possible infectious donor.

In the 2008 experiment (Experiment 2), potential susceptible mice were quarantined for 3 weeks in separate plastic mouse boxes in a temporary, locked, air-conditioned, quarantine facility that was separate from any other structures and had highly restricted access. The only personnel to approach or enter the facility were trained technicians wearing respirators with high-efficiency particulate air filters, eye protection, and protective outer clothing. Similar temporary structures and protective equipment have been used by CDC researchers responding to hantavirus disease outbreaks since the 1990s (see [[5](#_ENREF_5)]). Seventeen mice whose blood samples were positive for SNV RNA or antibody upon first capture were rereleased at the capture site. The mice whose blood was negative for SNV RNA and antibody were individually housed in separate plastic mouse boxes in the quarantine facility; their blood was retested approximately 2 weeks (14–16 days) post-capture and 3.5 weeks (25 days) post-capture before release into enclosures. Of the 54 quarantined individuals, 3 became SNV antibody or RNA positive during the first 2 weeks of the quarantine period. No mice became SNV antibody or RNA positive past the 2-week mark of quarantine. One of the 3 seroconverters was returned to where it had been captured. The 2 other seroconverters were used as donor mice. The rest of the donor mice were chosen based on SNV RNA and seroconversion data. We recaptured some of the positive mice that we had released prior to the quarantine of potential susceptible animals, and chose donors who were SNV RNA-positive and/or whose antibody titers had increased 4–16 fold during the 2-week period since their last capture.

**1.4 Statistical analyses and variables**

We report transmission incidence per each experiment (= season), and transmission incidence per density treatment during each experiment. Incidence of transmission was the number of transmission events divided by the sum of the number of weeks each mouse was in the experiment and susceptible to infection (see [[6](#_ENREF_6)]). By using these rates (=incidence), we control for the number of mice per treatment and experiment, and for varying durations of the experiments. We calculated rate ratios and confidence intervals for transmission incidence, and compared all pairs of seasons for statistically significant differences. We do not report rate ratios of transmission by density treatment, because the low-density treatment group had zero transmission events in 2 of the 3 density experiments. We used a test for differences in proportions to analyze both seasonal incidence and incidence by density treatment, although the data did not meet the typical sample size criteria for stable performance. Because of the very low number of transmission events and small samples sizes of our experiments, we also used Fisher’s exact two-tailed test to make pairwise comparisons of proportions of TE mice over all susceptible mice between all pairs of experiments (=seasons) and between all density treatments within each experiment.

In situations in which we made comparisons of proportions between dates, seasons, or experiments in our data analyses, we did not use Bonferroni corrections for our individual tests. Applying this type of correction increases the likelihood of type II errors, and we agree with those who feel that these corrections should not be used when assessing evidence about specific hypotheses [[7](#_ENREF_7),[8](#_ENREF_8)].

Where biweekly vs weekly sampling frequency might influence the values of the variables measured (i.e., comparing 2007 to 2008 data), we used only biweekly data from 2008. When the timing of data collection may have influenced variables (e.g., wound presence and absence, number of new wounds and reproductive status), we compared data collected at similar time periods (2 weeks post release into enclosures) for between-experiment comparisons and also pooled these data from all four experiments for any overall analyses.

**References**

1. Kaufman GA, Kaufman DW (1989) An Artificial Burrow for the Study of Natural Populations of Small Mammals. Journal of Mammalogy 70: 656.

2. Feldmann H, Sanchez A, Morzunov S, Spiropoulou CF, Rollin PE, et al. (1993) Utilization of autopsy RNA for the synthesis of the nucleocapsid antigen of a newly recognized virus associated with hantavirus pulmonary syndrome. Virus Research 30: 351-367.

3. Schountz T, Calisher CH, Richens TR, Rich AA, Doty JB, et al. (2007) Rapid field immunoassay for detecting antibody to Sin Nombre virus in deer mice. Emerging Infectious Diseases 13: 1604-1607.

4. Chomczynski P (1993) A reagent for the single-step simultaneous isolation of RNA, DNA and proteins from cell and tissue samples. BioTechniques 15: 532-534, 536-537.

5. Mills JN, Childs JE, Ksiazek TG, Peters CJ, Velleca WM (1995) Methods for trapping and sampling small mammals for virologic testing. Atlanta: U. S. Department of Health and Human Services. 61-61 p.

6. Mills JN, Ellis BA, McKee KT, Calder¢n GE, Maiztegui JI, et al. (1992) A longitudinal study of Jun¡n virus activity in the rodent reservoir of Argentine hemorrhagic fever. American Journal of Tropical Medicine and Hygiene 47: 749-763.

7. Nakagawa S (2004) A farewell to Bonferroni: the problems of low statistical power and publication bias. Behavioral Ecology 15: 1044-1045.

8. Perneger TV (1998) What's wrong with Bonferroni adjustments. BMJ 316: 1236-1238.
